# Survival After Adrenalectomy for Metastatic Hepatocellular Carcinoma: A 25-year Institutional Experience

**DOI:** 10.1007/s00268-020-05909-0

**Published:** 2020-12-22

**Authors:** JI. Staubitz, M. Hoppe-Lotichius, J. Baumgart, J. Mittler, H. Lang, TJ. Musholt

**Affiliations:** 1 Section of Endocrine Surgery, Department of General, Visceral and Transplantation Surgery, University Medical Center, Johannes Gutenberg-University Mainz, Langenbeckstraße 1, 55131 Mainz, Germany; 2 Department of General, Visceral and Transplantation Surgery, University Medical Center, Johannes Gutenberg-University Mainz, Mainz, Germany

## Abstract

**Background:**

Extrahepatic manifestation of hepatocellular carcinoma (HCC) is rare and primarily affects lung, lymph nodes and bone. Metastases to the adrenal glands are relatively infrequent. This 25-year institutional experience aimed for an analysis of factors influencing survival in patients undergoing surgery for HCC adrenal metastasis.

**Methods:**

A retrospective analysis of the institutional database of the Clinic for General-, Visceral- and Transplantation Surgery of the University Medical Center Mainz, Germany, was performed. Patients who underwent surgery for HCC adrenal metastases from January 1995 to June 2020 were included. Pre-, peri- and postoperative factors with potential influence on survival were assessed.

**Results:**

In 16 patients (14 males, two females), one bilateral and 15 unilateral adrenalectomies were performed (13 metachronous, three synchronous). Thirteen operations were carried out via laparotomy, and three adrenalectomies were minimally invasive (two laparoscopic, one retroperitoneoscopic). Median overall survival (after HCC diagnosis) was 35 months, range: 5–198. Median post-resection survival (after adrenalectomy) was 15 months, range: 0–75. Overall survival was longer in patients with the primary HCC treatment being liver transplantation (median 66 months) or liver resection (median 51 months), compared to only palliative intended treatment of the primary with chemotherapy (median 35 months) or local ablation (median 23 months).

**Conclusions:**

Surgery is a feasible treatment option for patients with adrenal metastases originating from HCC. In patients who underwent adrenalectomy for HCC adrenal metastasis, overall survival was superior, if primary HCC treatment was potentially curative (liver transplantation or resection).

## Introduction

Surgery is a therapeutic option to treat adrenal metastases of different tumor entities. For isolated adrenal metastases with metachronous onset in particular, adrenalectomy was observed to improve overall survival [1–3]. Still, survival is essentially influenced by the underlying tumor entity. Therefore, the literature recommends a careful selection of patients, who may benefit from adrenalectomy for metastatic disease [2, 4]. An individually tailored concept, resulting from a multidisciplinary tumor board discussion, should be the basis for the decision to perform surgery for adrenal metastases [5]. Tumor entities which are frequently observed to develop adrenal metastases are non-small cell lung cancer and malignant melanoma [1, 4, 6]. In rarer cases, adrenal metastases deriving from hepatocellular carcinoma (HCC) are registered [7–9]. Whereas extrahepatic HCC manifestation can generally be expected in up to 39.1% in the course of disease, the incidence rate of extrahepatic metastases after medical treatment of the primarius was observed to be approximately 2.5% per year [10–12]. The most common sites of extrahepatic HCC metastases are lung (39.5–53.1% of HCC patients with extrahepatic manifestation), lymph nodes (29.6–34.2%) and bone (25.4–43.2%) [10, 13–19]. In comparison, metastases to the adrenal glands are relatively infrequent (8.0–19.1% of HCC patients with extrahepatic manifestation) [12–16]. Due to the small number of cases, most studies addressing HCC with adrenal metastasis—even if performed by specialized centers for liver disease—refer to restricted patient cohorts (Table 1). The aim of this study was to report a 25-year institutional experience with surgery for adrenal metastasis deriving from hepatocellular carcinoma and to assess potential factors influencing patient survival, including different oncological treatment concepts.

**Table 1:** Studies on adrenalectomy for adrenal metastases deriving from hepatocellular carcinoma (patient count ≥ 5), published in the recent decade (2000–2020)

Authors	Year	Study period [years]	Size of underlying HCC cohort [*N*]	Patients with HCC adrenal metastasis [*N*]	Patients with adrenal metastasis undergoing adrenalectomy [*N*]	Median overall survival in patients with adrenalectomy for HCC metastasis [months]	Median post-resection survival after adrenalectomy for HCC metastasis [months]	Cumulative survival at 5 years after adrenalectomy [per cent]
Teegen et al. [15]	2018	10	1293	10	8	109.5, 126^a^	69, 81^a^	n/a
Ha et al. [30]	2014	15	5356	n/a	26	n/a	n/a	20.3, 85.7^a^
Park et al. [11]	2007	14	11770	45	5	n/a	21.41	n/a
Momoi et al. [31]	2002	13	793	24	13	n/a	n/a	25

^a^result for patients with initial HCC treatment being liver transplantation

n/a = not assessed

## Materials and methods

### Patients

A retrospective analysis of the institutional database of the Clinic for General-, Visceral- and Transplantation Surgery of the University Medical Center Mainz, Germany, was performed. All patients who underwent adrenalectomy with histologically proven metastasis of HCC to the adrenal gland from January 1995 to June 2020 were included. Metastases detected at ≤ 6 and > 6 months after treatment for the primary tumor were classified as synchronous and metachronous, respectively.

The indication to perform adrenalectomy was evaluated for the individual patient after discussion of the case in an interdisciplinary tumor board conference, taking into account current radiological imaging and clinical course of disease. Surgery was performed either with curative intention, or to control stable (i.e., therapeutically controlled) disease with singular progression of adrenal metastasis.

### Parameters of assessment

Basic patient characteristics and perioperative data were assessed. These included histopathological parameters (side of the adrenal tumor, tumor size), surgical parameters (surgical technique, resection extent: isolated adrenalectomy vs. extended adrenalectomy, duration of surgery), perioperative parameters (length of hospital stay) and postoperative follow-up (type of primary treatment for HCC, overall oncological concept, survival: post-resection survival and overall survival).

**Fig. 1:**
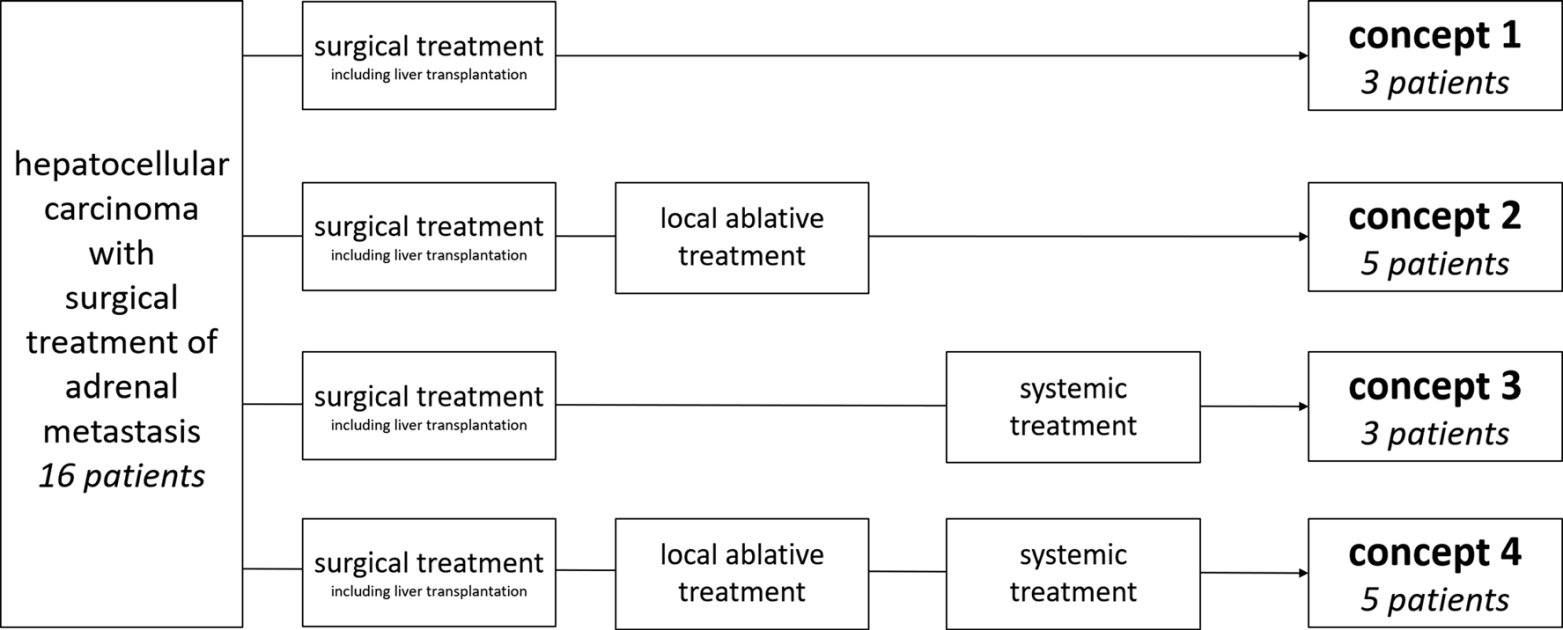
Different overall treatment concepts for metastatic hepatocellular carcinoma. Frequent overall treatment concepts were surgery combined to local ablative treatment (five of 15 patients) and a combination of surgical, local ablative and systemic treatment (five of 15 patients). Surgical treatment only (three patients) and a combination of surgical and systemic treatment (three patients) were less common concepts. Surgical treatment comprises cases of liver transplantation.

Resection extent was defined as “extended”, if the adrenalectomy included the resection of adjacent organs and/or lymphadenectomy. “Post-resection survival” was defined as the time from adrenalectomy up to the date of the last follow-up or death. “Overall survival” was defined as the time from initial HCC diagnosis to last follow-up or death. Oncological treatment concepts were categorized according to Fig. 1.

### Data analysis

Data were documented and described using Microsoft Excel (Microsoft Corporation, Redmond, USA). Categorical variables are presented as numbers and per cent, and continuous variables as median with range.

## Results

In the analyzed time period from January 1995 to June 2020, 3000 patients were treated for the diagnosis of hepatocellular carcinoma at the University Medical Center Mainz. Of these, 531 underwent liver resection in the course of treatment. Three hundred forty-five patients were treated with liver transplantation. The remaining patients received local ablative treatment (transcatheter arterial chemoembolization (TACE), percutaneous radiofrequency ablation, percutaneous ethanol injection therapy (PEIT) or radiotherapy) and systemic treatment (including sorafenib, pembrolizumab, ramucirumab and tamoxifen), alone or in combination. Sixteen patients with HCC adrenal metastases were referred to our surgical department for adrenalectomy in the course of disease.

### Basic patient characteristics and perioperative parameters

Of 16 adrenalectomies in total, the majority were performed in male patients (14 males, two females). Thirteen patients had metachronous onset of adrenal metastasis, and three were synchronous. Median patient age at the time of surgery for adrenal metastasis was 61 years (range: 50–84, Table 2). The median time interval from HCC diagnosis to diagnosis of adrenal metastasis was 22 months (range: 0–132, Table 2). Median tumor size was 7.6 cm (range: 1.5–20.5). In all but one cases, unilateral adrenalectomy was performed. One patient, who underwent bilateral adrenalectomy for bilateral adrenal metastases (1.5 cm and 0.7 cm), the larger tumor diameter was counted. In the case of bilateral adrenalectomy, due to the resulting lack of endogenous corticosteroid synthesis, a postoperative substitution with cortisol (35 mg per day, divided into three doses respecting the circadian rhythm) and fludrocortisone (0.1 mg per day) was necessary. Thirteen patients underwent open surgery, whereas three were operated using minimally invasive approaches (two laparoscopic, one retroperitoneoscopic, Table 3). Minimally invasive approaches were chosen for tumors with a smaller median diameter of 2.5 cm (range: 1.5–3.5). Median operation time was 187 min (range: 85–293) and median hospital stay 10 days (range: 3–110, Table 3). In all cases, R0 status were achieved. Median post-resection survival after adrenalectomy was 15 months (range: 0–75 months) and median overall survival 35 months (range: 5–198, Fig. 2).

**Table 2:** Basic patient parameters including subgroup analysis for liver transplantation

	Total (*n* = 16)	Liver transplantation (*n* = 5)	No liver transplantation (*n* = 11)
*Basic parameters*			
Patient sex Male sex (*N*) Female sex (*N*)	14 2	4 1	10 1
Patient age at the time of HCC diagnosis (years; median, range)	58, 48–82	49, 48–69	66, 48–82
Interval from HCC to adrenal metastasis (months; median, range)	22, 0–132	24, 20–132	13, 0–93
MELD score (at the time of adrenalectomy) (median, range)	9, 5–12	8, 5–9	9, 6–12
Patient age at the time of adrenal metastasis (years; median, range)	61, 50–84	51, 50–77	66, 50–84
Onset of metastasis Synchronous onset adrenal metastasis (*N*) Metachronous onset adrenal metastasis (*N*)	3 13	0 5	3 8
*Histopathological parameters adrenal metastasis*			
Tumor size (cm; median, range)	7.6, 1.5–20.5	7.5, 2.5–8	8, 1.5–20.5
Affected side Tumor left adrenal gland (*N*) Tumor right adrenal gland (*N*) Tumor bilateral (*N*)	8 7 1	2 3 0	6 4 1
*Follow-up*			
Palliative adrenalectomy—not tumor free after resection (*N*) Recurrence after adrenalectomy < two years after adrenalectomy (*N*) ≥ two years after adrenalectomy (*N*) Localization of recurrence Lung (*N*) Contralateral adrenal gland (*N*) Peritoneal (*N*) No recurrence documented after adrenalectomy	4 6 4 2 5 1 1 6	1 3 1 2 3 1 0 1	3 3 3 0 2 0 1 5
Median overall survival (months; median, range)	35, 5–189	96, 35–189	33, 5–93
Median post-resection survival (months; median, range)	15, 0–75	57, 11–75	13, 0–29
*Oncological concept*			
Primary treatment for HCC Liver surgery (*N*) Liver transplantation (*N*) Local ablation (*N*) Systemic treatment (*N*)	7 4 4 1	1 4 0 0	6 0 4 1
Overall oncological concept Concept 1 according to Fig. 1 (*N*) Concept 2 according to Fig. 1 (*N*) Concept 3 according to Fig. 1 (*N*) Concept 4 according to Fig. 1 (*N*)	3 5 3 5	0 0 1 4	3 5 2 1

**Table 3:** Surgical and perioperative parameters including subgroup analysis for liver transplantation

	Total (*n* = 16)	Liver transplantation (*n* = 5)	No liver transplantation (*n* = 11)
Intention of adrenalectomy Curative intention (*N*) Palliation (*N*)	13 3	4 1	9 2
Resection technique for adrenalectomy Open surgical approach (*N*) Laparoscopic (*N*) Retroperitoneoscopic (*N*)	13 2 1	4 0 1	9 2 0
Resection extent Isolated adrenalectomy (*N*) Extended adrenalectomy (*N*)	12 4^a^	4 1^b^	8 3^c^
Duration of surgery (min; median, range)	187, 85–293	158, 85–293	188, 112–275
Duration of hospital stay (days; median, range)	10, 3–110	9, 4–14	10, 3–110

^a^*Resected structures* liver segment 6/7 + biopsy liver segment 4 + partial resection of the diaphragm/resection of tumor thrombus in the inferior cava vein + liver segment 1/atypical resection of liver segment 3 + lymphadenectomy at the mesenteric artery/lymphadenectomy liver hilus + partial resection of the diaphragm

^b^*Resected structures* lymphadenectomy liver hilus + partial resection of the diaphragm

^c^*Resected structures* liver segment 6/7 + biopsy liver segment 4 + partial resection of the diaphragm/resection of tumor thrombus in the inferior cava vein + liver segment 1/atypical resection of liver segment 3, lymphadenectomy at the mesenteric artery

**Fig. 2:**
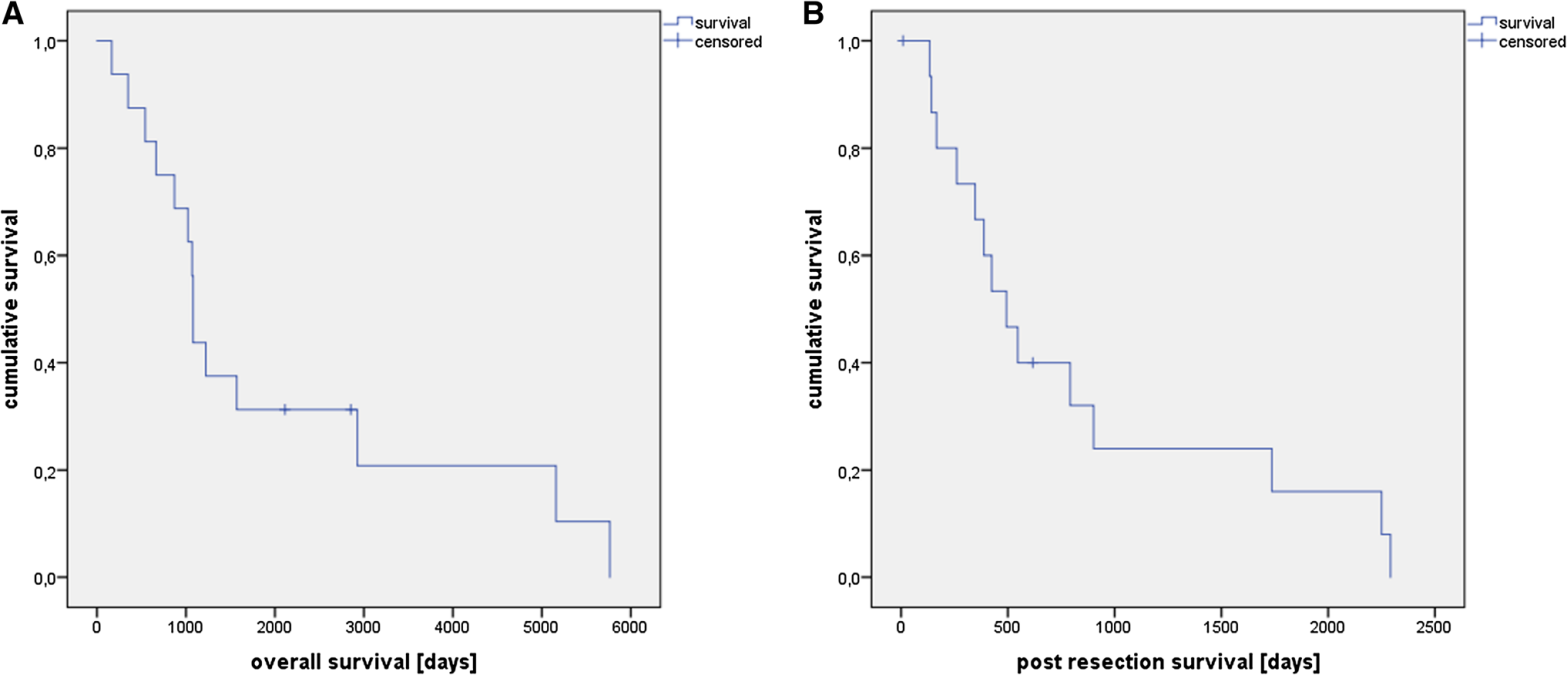
Kaplan–Meier survival curves for **a** overall survival and **b** post-resection survival. Due to a high portion of metachronous metastases, median post-resection survival was shorter than median overall survival (15 vs. 35 months).

### Influence of basic patient characteristics and perioperative parameters on survival

Metachronous onset of adrenal metastasis was associated with longer overall survival, compared to synchronous disease (median 40 months vs. 33 months). Post-resection survival was not influenced by the onset. Neither the surgical technique chosen for adrenalectomy nor resection extent (isolated vs. extended adrenalectomy) played a role for survival in the present analysis.

### Distribution of primary HCC treatment, overall treatment concepts and influence on survival

The primary treatment for HCC was surgery in 11 cases: in seven patients, liver resection was performed, and four patients received liver transplantation. One patient, who was initially treated with liver resection, received liver transplantation in the further course. There was a tendency of patients who received liver transplantation to develop adrenal metastasis later than those without liver transplantation (median 24 vs. 13 months, Table 2). In four patients, primary treatment was local ablation (Table 2). Only in one case, systemic treatment was the primary therapy approach for underlying HCC. In this case, sorafenib was applied. Within the combined overall treatment concept, which included surgery and systemic treatment (concept 3, Fig. 1), all patients received sorafenib and one patient received pembrolizumab and ramucirumab in the course of disease. In case of the combined surgical, local ablative and systemic treatment (concept 4, Fig. 1), systemic treatment was performed with sorafenib in all cases, and in three cases with rapamycin in the further course.

The choice of primary treatment influenced overall survival in patients with HCC and surgically treated adrenal metastasis in the course of disease: if primary treatment was liver resection, a median survival of 51 months was observed, for liver transplantation 66 months, for systemic treatment 35 months and for local ablation 23 months. Similarly, post-resection survival after adrenalectomy was tendentially longer for patients, who initially underwent surgical treatment for HCC (median post-resection survival for liver resection/transplantation: 16 months versus 8 months in the case of local ablation/systemic treatment).

Frequent overall treatment concepts for HCC were surgery plus local ablative treatment (five of 16 patients) and a combination of surgical, local ablative and systemic treatment (five of 16 patients). Surgical treatment only (three patients) and a combination of surgical and systemic treatment (three patients) were less common treatment strategies (Fig. 1, Table 2). Neither overall survival nor post-resection survival was influenced by the overall oncological treatment concept according to Fig. 1. Yet, overall survival and post-resection survival were tendentially longer, if the overall treatment concept included liver transplantation (median overall survival in the case of liver transplantation: 96 versus 33 months; median post-resection survival in the case of liver transplantation: 57 vs. 13 months).

## Discussion

Adrenal metastasis originating from HCC is rare. In a cohort of 11770 HCC patients treated at Yonsei University Medical Center, Seoul, Korea, Park et al. observed 45 individuals with adrenal metastasis (0.4%), whereas Teegen et al. reported ten patients with HCC adrenal metastasis in a cohort of 1293 patients treated with either liver resection (990 patients) or liver transplantation (303 patients) for underlying HCC at Charité University Medical Center, Berlin, Germany (0.8%) [11, 15]. In this sense, the seemingly small number of cases in the current cohort undergoing adrenalectomy for HCC adrenal metastasis (16 patients) represents a noteworthy collective of 25 years of surgical practice in our clinic. The prognosis of untreated HCC adrenal metastasis is poor, with overall survival described to be expectedly 6 months [11, 20, 21]. Currently, there are no definitive guidelines of how to treat adrenal metastases originating from HCC [11]. Potential treatment options are surgery, local ablative treatment (transcatheter arterial chemoembolization (TACE), percutaneous radiofrequency ablation, percutaneous ethanol injection therapy (PEIT) or radiotherapy) and systemic treatment (sorafenib), which should be chosen according to the overall condition of the individual patient concerned [11, 13, 19, 20, 22–24]. Adrenalectomy was shown to be associated with significantly longer survival, compared to other treatment options [11]. In the present cohort of patients with surgically treated HCC adrenal metastases, the choice of the primary therapy for HCC had an influence on the outcome. Overall survival was longer, if HCC was primarily treated with liver transplantation (median 66 months) or liver resection (median 51 months), compared to other primary therapy approaches (systemic treatment: median 35 months, local ablation: 23 months). Similarly, post-resection survival illustrated a more favorable outcome for surgically treated patients (liver resection or transplantation). Another factor which influenced overall survival was the onset of HCC adrenal metastasis: metachronous onset was associated with tendentially longer overall survival. In the present cohort, synchronous adrenal HCC metastasis was observed in three cases (all three cases received different primary HCC treatment: systemic treatment with sorafenib, local ablative treatment or liver resection with simultaneous adrenalectomy). The literature described that liver transplantation as a primary therapy for HCC was associated with later onset of metastasis to the adrenal gland, when compared to different primary treatment concepts [15, 25]. Also, in the present cohort, the onset of adrenal metastasis was later, if the overall treatment concept included liver transplantation (median 24 months vs. 13 months). Yet, most patients of the present cohort received multimodal treatment in the course of disease (Fig. 1). Combinations of surgical treatment and local ablative treatment as well as combinations of these approaches with additional systemic treatment were common. The indication to perform surgery for adrenal metastases in the present cohort was based on interdisciplinary tumor board conference discussion and was either curative (13 of 16 cases) or to treat otherwise stable (i.e., therapeutically controlled) disease with singularly progressive adrenal metastasis (three of 16 cases). In this study, post-resection survival after adrenalectomy for adrenal HCC metastasis was 15 months in the entire group. Park et al. [11] observed a superior post-resection survival after adrenalectomy of 21 months. In comparison, Teegen et al. [15] reported a median post-resection survival of 69 months after adrenalectomy for metastatic HCC. Whereas the median overall survival in the present cohort of patients who underwent surgery for HCC adrenal metastasis was 35 months, Teegen et al. [15] observed a median overall survival of 110 months. If patients had received liver transplantation, median overall survival in the cohort reported by Teegen amounted to 126 months [15]. The inclusion of only recently operated patients with short follow-up accounts for the shorter overall survival times in our study. In addition, three cases of palliative adrenalectomy (19%) were included. Finally, changes in the surgical and oncological management over the past 25 years influenced the presently reported results, whereas the operations documented by Teegen et al. [15] were collected from 2005 onward, resulting in a more homogeneous patient group.

Depending on tumor size and surgical expertise, minimally invasive techniques are considered suitable to resect locally confined adrenal metastases [5, 26, 27]. In patients suffering from HCC, who in many cases harbor liver cirrhosis, portal hypertension and a potentially compromised blood coagulation, the use of minimally invasive techniques can be complicated. In the present cohort, two adrenalectomies were performed laparoscopically and one was resected via a retroperitoneoscopic approach. Minimally invasive techniques were chosen for tumors with a median diameter of 2.5 cm, whereas larger tumors (> 6 cm) prompted open adrenalectomy. Similarly, other centers reported the successful application of minimally invasive surgery for HCC adrenal metastasis [15, 28]. Due to the extended interval of this retrospective study (25 years), changes in the surgical management and the primary choice of techniques might have influenced the observed distribution of surgical techniques: in the early study period, also for smaller tumors, open surgery was chosen for adrenalectomy. However—as a major drawback of the study—the absolute number of patients is associated with a limited statistical power, which has to be taken into account when considering the results. Furthermore, the selection of patients for adrenalectomy includes a bias with respect to tumor burden and comorbidities. Yet, a prospective randomized trial that investigates the benefits of adrenalectomy in these rare subgroups of patients will not be possible.

The current literature illustrates that adrenalectomy is a safe treatment for adrenal metastases originating from HCC [7, 11, 15, 29]. The presented results support this impression. In this selected cohort of patients who underwent surgery for HCC adrenal metastases, overall survival was superior, if the primary treatment for underlying HCC was liver transplantation or liver resection (compared to chemotherapy or local ablation) and if the onset of disease was metachronous. Also, post-resection survival after adrenalectomy was tendentially longer in patients who initially received surgical and therefore potentially curative HCC therapy (liver transplantation or liver resection).

Therefore, adrenalectomy can be considered in selected patients with underlying metastatic HCC and should receive particular attention in patients with metachronous onset of disease and in patients with primarily performed liver transplantation or liver resection. It can be performed via minimally invasive approaches, dependent on tumor size and basic patient conditions.
